# Investigation of Transcript Variant 6 of *TPD52L2* as a Prognostic and Predictive Biomarker in Basal-Like MDA-MB-231 and MDA-MB-453 Cell Lines for Breast Cancer

**DOI:** 10.1155/2022/7078787

**Published:** 2022-08-29

**Authors:** Xin Zhang, Daniel O'Brien, Xiaohui Zhang

**Affiliations:** ^1^Department of Spine Surgery, Weifang People's Hospital, Weifang, Shandong 261000, China; ^2^Key Laboratory of Human Spine Biomechanics of Weifang City, Weifang, Shandong 261000, China; ^3^Bioinformatics Core, Mayo Clinic, Rochester, MN 55905, USA; ^4^Department of Breast Surgery, Peking Union Medical College Hospital, Chinese Academy of Medical Science, Beijing 100730, China

## Abstract

**Background:**

Basal-like breast cancer (BLBC) exhibits worse pathological features than other breast cancer subtypes, and patients diagnosed with BLBC have short disease-free and overall survival times. Thus, the identification of novel biomarkers and therapeutic targets for BLBC is of upmost importance. Although TPD52L2 is upregulated in multiple cancers, little is known about its roles in BLBC.

**Methods:**

RNA levels were analyzed between breast cancer tissues and paired adjacent normal tissues using RNA-seq data from The Cancer Genome Atlas (TCGA). TPD52L2 stable knockdown and inducible knockout cell lines were established using basal-like MDA-MB-231 and MDA-MB-453 cell lines. Cell proliferation assays *in vitro* and tumor growth analysis *in vivo* were performed to determine the function of TPD52L2 during BLBC progression. Transwell assays were used to estimate the regulatory effect of TPD52L2 on BLBC cell migration. The expression profile of all *tpd52l2* transcripts was analyzed to assess the functional protein isoform. Association of transcript variant 6 (V6) expression with pathological parameters was carried out using the clinical data of the BRCA cohort.

**Results:**

We identified V6 of TPD52L2 as a novel biomarker and regulator of BLBC progression. TPD52L2 is upregulated in BLBCs and associated with patient outcomes. TPD52L2 knockdown suppresses tumor growth, and V6 correlates with cancer-related phenotypes in BLBC. Clinical data further proved that V6 is associated with different pathological features, such as pathological stage and pathological tumor status, and independently predicts patient outcomes and responses to therapies.

**Conclusions:**

Our findings demonstrate that V6 of TPD52L2 is a novel biomarker for BLBC patients. V6 promotes cell proliferation and migration and has marked oncogenic roles in determining the malignant phenotypes of BLBC.

## 1. Introduction

Breast cancer (BC) is one of the most lethal diseases and led to almost 685,000 female deaths in 2020 worldwide [1]. It is a heterogeneous disease with diverse molecular alterations and cellular components. The PAM50 signature is used to divide breast cancers into luminal A, luminal B, Her 2-enriched, basal-like, and normal-like subtypes. Compared to other subtypes, basal-like breast cancer (BLBC) exhibits worse clinical and pathological features, such as poorer differentiation, higher proliferation capacity, increased stemness and metastasis potential, and more prominent lymphocyte infiltration. Patients diagnosed with BLBC generally have short disease-free and overall survival times [2]. Most BLBC cases are triple-negative breast cancer (TNBC) (negative expression of estrogen receptor (ER), progesterone receptor (PR), and Her2 receptor). Endocrine and target molecular therapies have limited effects on this malignant subtype [3]. Genomic and transcriptomic analyses contribute to the understanding of the molecular basis and malignant phenotypes of cancers. Many biomarkers have been identified and have decreased the lethality of BLBC in recent years. A signature of 80 genes distinguished two basal-like subgroups with different clinical features. This signature was associated with the cancer immune response and epithelial-mesenchymal transition [4]. To date, biomarkers that are associated with pathological features and can predict clinical outcomes have been limited in BLBC. Therefore, the identification of new biomarkers and therapeutic targets for BLBCs and the demonstration of their molecular mechanisms will lead to better management of this aggressive disease.

The tumor protein D52-like family is a group of coiled coil proteins. This family consists of TPD52, TPD52L1 (TPD53), TPD52L2 (TPD54), and TPD52L3 (TPD55), which are highly expressed in multiple cancers. TPD52 was shown to be overexpressed in breast cancer and prostate cancer and identified as a candidate oncogene [5, 6]. TPD52 overexpression increased the storage of fatty acids in triglycerides in cultured cells and formed more lipid droplets after oleic acid supplementation [7]. In oral squamous cell carcinoma (OSCC), TPD52 was elevated under hypoxic conditions in a HIF-independent manner and promoted cell proliferation and survival [8]. It was recently validated that immunohistochemistry (IHC) of TPD52 had important prognostic values for group 3/4 medulloblastomas [9]. These data suggested that inhibition of TPD52 could contribute to cancer treatment.

TPD52L2 is another abundant member of the tumor protein D52-like family. Proteomic analysis revealed that TPD52L2 was one of the most abundant proteins in HeLa cells, ranking 180th out of 8,804 proteins, with an estimated 3.3 × 10^6^ copies per cell [10]. TPD52L2 is involved in a new class of intracellular transport vesicles: intracellular nanovesicles (INVs). It can interact with Rab GTPases and bind directly to INVs to regulate the trafficking of specific cargos with dileucine sorting motifs [11]. However, there are limited studies on its cellular functions, molecular mechanisms, and clinical significance in different types of cancers. In this study, we evaluated TPD52L2 expression and its impact on patient outcomes in a breast cancer cohort from The Cancer Genome Atlas (TCGA) dataset. We found that TPD52L2 is upregulated in BLBC and is notably associated with the outcomes of patients with BLBC and lymph node-positive BLBC. TPD52L2 knockdown had an inhibitory effect on the growth of BLBC cells, and transcript variant 6 (V6) was identified to be translated into the oncogenic protein isoform. Clinical data further validated that V6 is linked to pathological features and predicts patient outcomes and therapy response. Our results established TPD52L2 as a novel biomarker in BLBCs and a potential therapeutic target.

## 2. Materials and Methods

### 2.1. TPD52L2 Expression Analysis in Tumors and Normal Tissues

Data for TCGA cohorts were downloaded from the UCSC Xena browser and analyzed [12]. The TPD52L2 expression data and patient survival data for 33 cancer types were obtained from TCGA. All the clinical parameters of breast cancer patients were downloaded from the BRCA cohort (http://xena.ucsc.edu/). Normal (GTEx samples) and tumor (TCGA samples) tissues were compared using resources from TCGA TARGET GTEx dataset in UCSC Xena [12, 13]. The overall survival of patients in the GSE96058 dataset was analyzed using the published resource Kaplan–Meier plotter (http://kmplot.com/) [14].

### 2.2. Cell Culture and Expression Plasmids

MDA-MB-231 and MDA-MB-453 cells were cultured in Dulbecco's modified Eagle's medium (DMEM, HyClone; Thermo Scientific) with 10% fetal bovine serum (FBS), 100 U/mL penicillin, and 100 *μ*g/mL streptomycin in 5% CO_2_-humidified incubators at 37°C. All cell lines were purchased from the American Type Culture Collection (ATCC) and free of mycoplasma contamination (tested by the vendor). Expression plasmids of ten transcript variants of *tpd52l2* were purchased from GenScript. Briefly, the cDNAs of ten transcript variants of *tpd52l2* were cloned into vector pcDNA3.1 with an HA tag linked to the C-terminus of the protein sequence. All constructs were confirmed by DNA sequencing.

### 2.3. TPD52L2 Stable Knockdown Cell Lines

*TPD52L2* short hairpin RNA (shRNA) plasmids were purchased from OriGene. The target sequences of shRNAs used in this study included GGAAGGGAGGTTGTCACTG, AGAAAGGTGCGGGATCCGA, and TGGCGCAGAGTGACAATTT. MDA-MB-231 cells were transfected with shRNA plasmids using Lipofectamine 2000 transfection reagents purchased from Invitrogen. The transfected cells were selected with 2 *μ*g/mL puromycin 36 h after transfection for 5 days. Clones were isolated and validated by Western blotting and sequencing of genomic DNA.

### 2.4. Cell Viability Assay

Viable cells were measured using Cell Counting Kit-8 (CCK-8, Dojindo) as previously described [15]. Briefly, 3000 cells per well were seeded in 96-well plates, and the medium was replaced every 3 days. Ten microliters of CCK-8 solution were added to each well containing 100 *μ*L of culture medium and incubated for 2 h at 37°C. The absorbance was measured at 450 nm using an ELISA plate reader. Cell proliferation was measured once per day for seven days.

### 2.5. Colony Formation Assays

In total, 1000 MDA-MB-231 cells or 2000 MDA-MB-453 cells per well were seeded in 6-well plates. The cells were cultured for 14 days, and visible colonies were formed. The cells were fixed with 4% paraformaldehyde solution and visualized by staining with 1% crystal violet. The colonies were counted. Each assay was performed in triplicate and repeated three times.

### 2.6. Migration Assay

Migration assays were performed on Transwell plates (Millipore). MDA-MB-231 cells (1 × 10^5^) and MDA-MB-453 cells (2 × 10^5^) were seeded on a polycarbonate membrane insert placed in a Transwell plate and cultured in DMEM without serum; then, DMEM containing 10% FBS was added to the wells of the plates [15]. After incubation at 37°C in a CO_2_ incubator for 18 h, the membrane was washed with phosphate-buffered saline (PBS), and the cells at the top of the membrane were wiped with cotton swabs. Cells that migrated to the bottom of the membrane were fixed with methanol, stained with 1% crystal violet, and counted in nine random fields at 200x magnification. Each assay was performed in triplicate and repeated three times.

### 2.7. Western Blotting

Proteins were extracted with SDS lysis buffer (50 mM Tris-HCl (pH 6.8), 10% glycerol, and 2% SDS) and quantified using the BCA protein assay reagent (Thermo Fisher). Extracts were loaded on a 12% SDS-PAGE gel, separated, and then electrophoretically transferred to a PVDF membrane (GE Healthcare) [15]. The membrane was blocked in 2% skim milk for 0.5 h at room temperature and then incubated overnight with the indicated antibodies at 4°C. The membrane was incubated with an HRP-IgG (Santa Cruz) secondary antibody for 1 h at room temperature. Chemiluminescence was detected using an ECL blot detection system (Santa Cruz). Vinculin (13901) and HA-tagged (3724) antibodies were purchased from Cell Signaling Technology Company. The TPD52L2 (11795-1-AP) antibody was purchased from Proteintech Company.

### 2.8. Hierarchical Clustering and Transcript Expression Analysis

The expression of ten *TPD52L2* transcript variants of 33 cancer types from TCGA dataset, which included information for a total of 9552 cancer tissues (containing 9185 primary cancers and 366 metastatic cancers of the SKCM cohort) (additional files), was downloaded from the UCSC Xena browser and analyzed by unsupervised hierarchical clustering (median value of transcript expression) using ClustVis at http://biit.cs.ut.ee/clustvis/ [12, 16].

### 2.9. Gene Structure and Protein Sequence Analyses

The exon/intron distributions were analyzed using GSDS software [17]. The mRNA, cDNA, and gene sequences of *TPD52L2* are listed in additional files. The amino acid sequences of ten protein isoforms of TPD52L2 were compared using Clustal Omega2 software with default parameters [18]. Protein sequences are listed in additional files.

### 2.10. TPD52L2 Inducible Knockout Cell Lines

Tetracycline-inducible lentiviral hEF1*α*-Blast-Cas9 nuclease particles and Edit-R lentiviral sgRNA particles were obtained from Dharmacon. The target sequence of the sgRNA was GCTCAGGGCTGAGCTTACCA. MDA-MB-453 cells were infected at an MOI of 5 according to the manufacturer's instructions. Briefly, MDA-MB-453 cells were infected with inducible Cas9 lentivirus, and 36 h after infection, cells were cultured in DMEM containing 10% tetracycline-free serum (HyClone) with 8 *μ*g/mL blasticidin S (Fisher Scientific) to select for stably expressing cells. Cells were passaged every 3 days for two weeks, and then, single clones were isolated and validated by Western blotting. Next, the inducible Cas9 clones were expanded and infected with sgRNA lentivirus. Cells were cultured in 2 *μ*g/mL puromycin (Fisher Scientific) for 5 days to obtain stable cell lines, and then, single clones were isolated and expanded. Dox (1 *μ*g/mL) hyclate (Fisher Scientific) was added to Tet-free medium for 2 days, and stably expressing cells were examined and selected by Western blotting.

### 2.11. *In Vivo* Tumorigenesis

Animal experiments were performed in accordance with a protocol approved by the animal care and use committees of Weifang People's Hospital. Five million tumor cells were resuspended in 0.1 mL phosphate-buffered saline and inoculated into the flanks of 6-week-old female athymic nude mice. Ten mice were injected in each group. Tumor growth was monitored every 3 days by measuring tumor diameters. Tumor width (*W*) and length (*L*) were measured, and tumor volume was calculated using the following formula: volume = (*W* × *L*)^2^/2. Mice were sacrificed 28 days after inoculation. The tumors were removed, photographed, and weighed, and the average weight of the tumors was calculated (^∗^*p* < 0.01).

### 2.12. Statistical Analysis

Statistical analysis was performed using GraphPad Prism 9. The results were statistically evaluated, and *p* < 0.05 was considered statistically significant. The Mann–Whitney test was used to compare the expression of TPD52L2 between cancer tissues and unpaired normal tissues, as well as in different subgroups. A paired *t*-test was used to analyze the difference in expression between cancer tissues and paired adjacent normal tissues. Fisher's exact test and Pearson's chi-squared test were used to determine the association between TPD52L2 (or transcript V6) expression and clinical parameters of breast cancer patients [19]. Survival data were analyzed using the Kaplan–Meier method with the log-rank test [19, 20].

## 3. Results

### 3.1. TPD52L2 Expression Is Upregulated in BLBC

**Figure 1:**
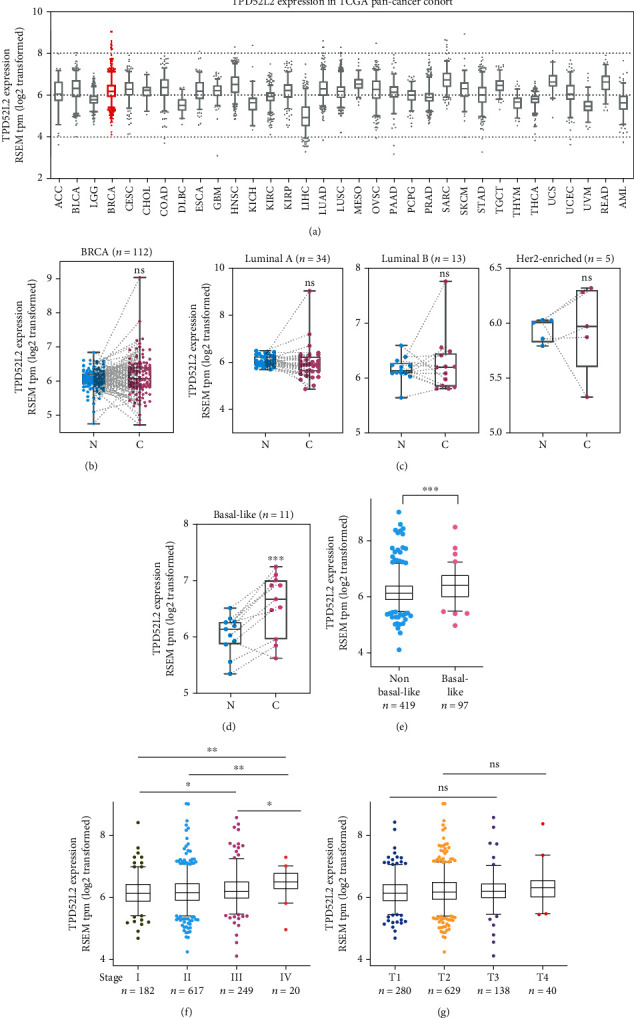
Expression analysis of TPD52L2 in breast cancer. (a) TPD52L2 expression in cancer tissues based on TCGA dataset. (b) Comparative analysis of TPD52L2 expression between cancer tissues and paired adjacent normal tissues from the BRCA cohort of TCGA. The difference was not significant (paired *t*-test). (c, d) TPD52L2 expression between cancer tissues and paired adjacent normal tissues in breast cancer patients with different PAM50 subtypes. (d) For BLBC patients, the difference between groups was significant (paired *t*-test, ^∗^*p* < 0.05). (e) TPD52L2 expression between non-BLBC and BLBC patients from the BRCA cohort. The difference between groups was significant (Mann–Whitney test). (f, g) Comparative analysis of TPD52L2 expression in breast cancer patients from the BRCA cohort: (e) stages I, II, III, and IV and (f) T1, T2, T3, and T4. The differences between groups were significant (Mann–Whitney test, ^∗^*p* < 0.05).

We found that TPD52L2 was highly expressed in the 1092 patients with primary breast cancer from TCGA BRCA cohort, with a median expression value of 6.177 (Figure 1(a)). However, no remarkable difference could be detected between 112 breast cancer tissues and paired adjacent normal tissues (Figure 1(b)). To expand our analysis, we investigated TPD52L2 expression in patients with luminal A, luminal B, and Her 2-enriched, and BLBCs (Figures 1(c) and 1(d)). As shown in Figure 1(d), TPD52L2 was upregulated in BLBCs, with a median value of 6.67, which was significantly higher than that in paired adjacent normal tissues, with a median value of 6.14 (*n* = 11, *p* < 0.001). For other subtypes, including luminal A, luminal B, and Her 2-enriched, TPD52L2 expression showed no difference between cancer tissues and paired adjacent normal tissues.

Importantly, TPD52L2 expression in 97 patients with BLBCs was much higher than that in 419 patients with non-BLBCs (Mann–Whitney test, *p* < 0.001) (Figure 1(e)). We further examined whether TPD52L2 expression was correlated with pathological stage and pathological tumor (pT) status. TPD52L2 expression increased significantly for patients with higher pathological stages compared to those with lower stages (Mann–Whitney test, stage IV *vs.* stage I: *p* = 0.0014; stage IV *vs.* stage II: *p* = 0.0048) (Figure 1(f)). The median expression value was 6.13 in patients with stage I disease and 6.51 in patients with stage IV disease. No significant correlation was found between TPD52L2 expression and tumor size (T1 *vs.* greater) (Figure 1(g)).

### 3.2. TPD52L2 Predicted the Prognosis of BLBC

**Figure 2:**
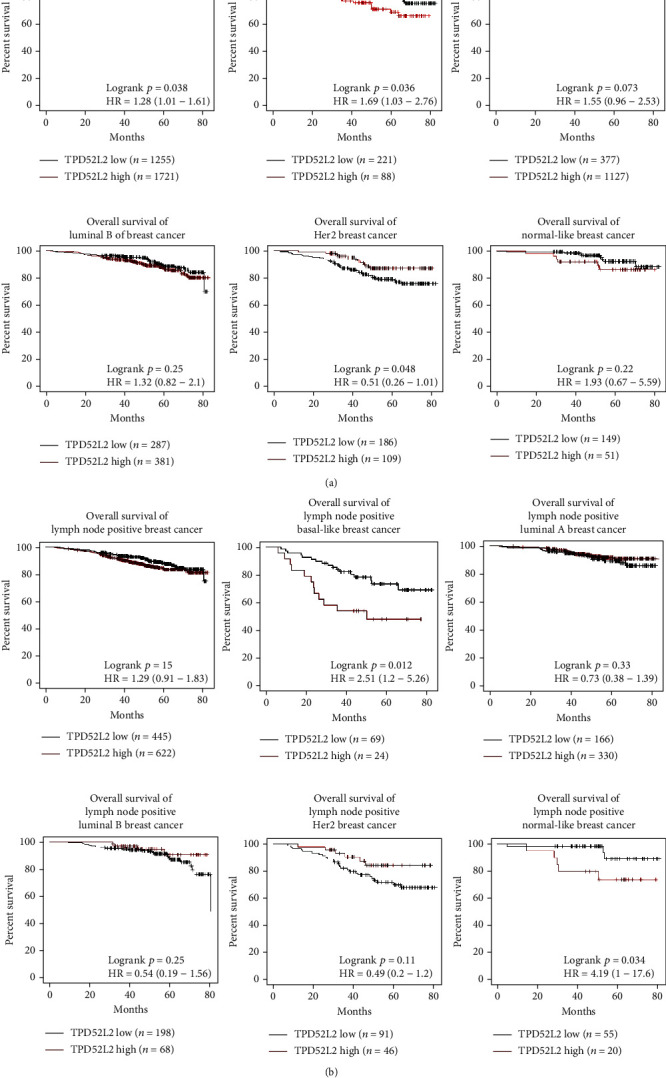
Kaplan–Meier curves indicating the OS of breast cancer patients with low and high TPD52L2 levels from the GSE96058 dataset [14]. (a) Kaplan–Meier curves of the OS of all breast cancer patients and patients with different PAM50 subtypes, including basal-like, luminal A, luminal B, Her2, and normal-like subtypes. (b) Kaplan–Meier curves of the OS of all lymph node-positive breast cancer patients and lymph node-positive patients with different PAM50 subtypes, including basal-like, luminal A, luminal B, Her2, and normal-like subtypes (log-rank test, ^∗^*p* < 0.05).

We analyzed the OS of patients from another breast cancer dataset, GSE96058 [14]. Patients with BLBC showed better overall survival in the TPD52L2 low-expression group than in the TPD52L2 high-expression group (Kaplan–Meier survival analysis with log-rank test, basal-like: *n* = 309, *p* = 0.036) (Figure 2(a)). Among the lymph node-positive BLBC patients, those in the TPD52L2 low-expression group lived longer than those in the TPD52L2 high-expression group (Kaplan–Meier survival analysis with log-rank test, basal-like: *n* = 93, *p* = 0.012) (Figure 2(b)). As for other PAM50 subtypes, the OS time of lymph node-positive patients was not different between the low-expression group and the high-expression group (Figures 2(a) and 2(b)).

**Figure 3:**
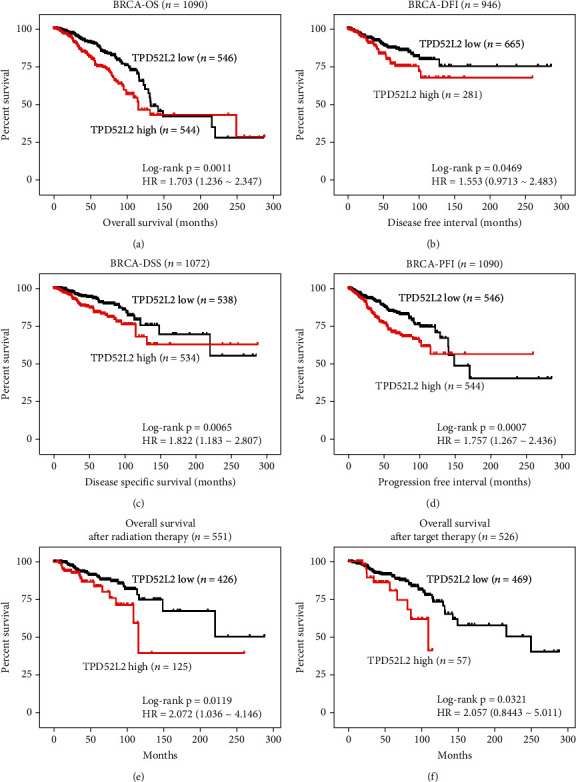
TPD52L2 impact on breast cancer patient survival and therapy response. (a–d) Kaplan–Meier curves indicate the OS, DFI, DSS, and PFI of breast cancer patients with low and high TPD52L2 levels from the BRCA cohort. (e, f) Kaplan–Meier curves indicate the OS of breast cancer patients with low and high TPD52L2 levels after treatment with radiation therapy or targeted therapy from the BRCA cohort (log-rank test, ^∗^*p* < 0.05).

We then analyzed whether TPD52L2 expression was correlated with the prognosis of breast cancer patients in the BRCA cohort. As shown in Figure 3(a), the median survival was 132 months for patients in the TPD52L2 low-expression group and 115 months for patients in the TPD52L2 high-expression group (Kaplan–Meier survival analysis with log-rank test, *n* = 1090, *p* = 0.0011) (Figure 3(a)). TPD52L2 expression was also correlated with disease-free interval (DFI), disease-specific survival (DSS), and progression-free interval (PFI). The outcomes were better for patients with low TPD52L2 expression than for patients with high TPD52L2 expression (Kaplan–Meier survival analysis with log-rank test, DFI: *n* = 946, *p* = 0.0469; DSS: *n* = 1072, *p* = 0.0065; and PFI: *n* = 1090, *p* = 0.0007) (Figures 3(b)–3(d)). Furthermore, we observed significant impacts of TPD52L2 on the overall survival of patients who received radiation therapy, and OS time decreased dramatically in the high TPD52L2 expression group (Kaplan–Meier survival analysis with log-rank test, *p* = 0.0119) (Figure 3(e)). Similarly, for patients who received targeted molecular therapy, those with low TPD52L2 levels received a greater survival benefit (median time: ~248 months) than those with high TPD52L2 levels (median time: ~109 months) (Kaplan–Meier survival analysis with log-rank test, *p* = 0.0321) (Figure 3(f)).

### 3.3. The Expression Profile of *tpd52l2* Transcript Variants in Breast Cancer

**Figure 4:**
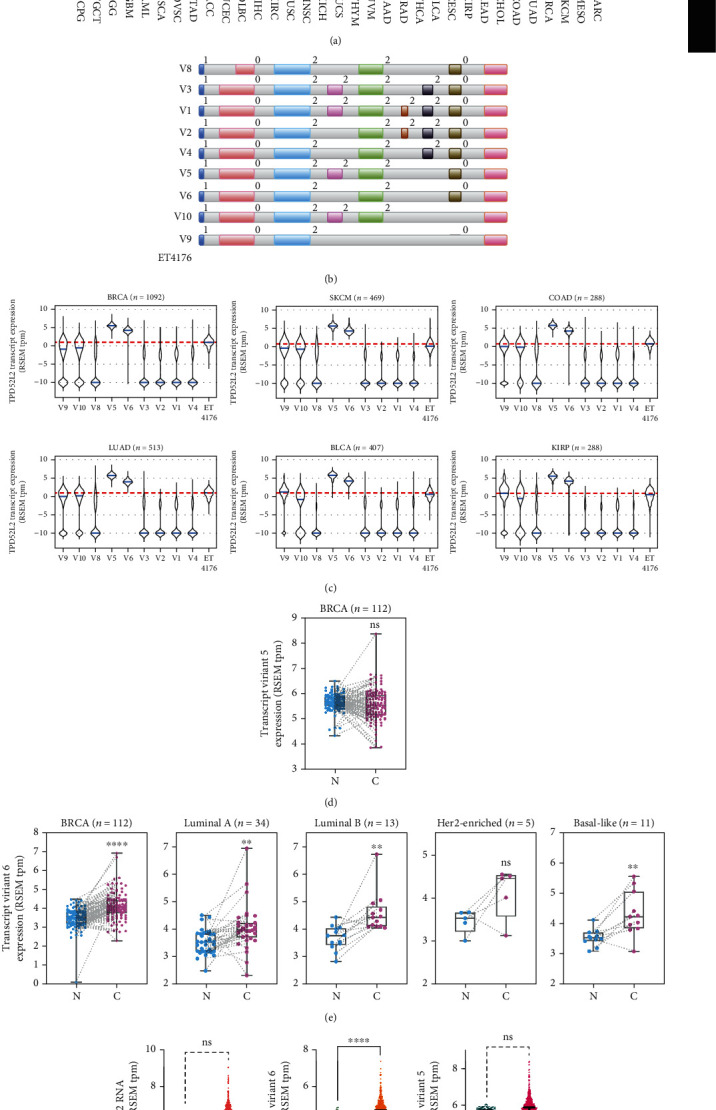
Expression profiles of *tpd52l2* transcript variants. (a) Heatmap of the expression of the ten transcript variants of the *tpd52l2* gene in patients from 33 cohorts of TCGA dataset (including 469 patients with primary and metastatic cancers from SKCM and 9083 patients with primary cancers from the other 32 cohorts). Cohorts were arranged by unsupervised clustering of median transcript expression. (b) Intron and exon distributions of each transcript variant of the *tpd52l2* gene. Colored bars represent exons, and white-gray bars represent introns. 0/1/2 represent the intron/exon phase. (c) Comparative analysis of *tpd52l2* transcript expression in patients with primary cancers from the BRCA, COAD, LUAD, BLCA, and KIRP cohorts and in patients with primary and metastatic cancers from the SKCM cohort of TCGA dataset. Blue lines are the median expression of each transcript. Red dashed lines indicate the cutoff value of one. (d) Expression of transcript variant 5 of *tpd52l2* between breast cancer tissues and paired adjacent normal tissues from the BRCA cohort of TCGA dataset. *n* = 112, no significant difference, paired *t*-test. (e) Expression analysis of transcript variant 6 of *tpd52l2* between breast cancer tissues and paired adjacent normal tissues grouped by PAM50 in the BRCA cohort (paired *t*-test, ^∗^*p* < 0.05). (f) Comparative analysis of the mRNA expression level of *tpd52l2* and the transcript expression levels of V5 and V6 between cancer tissues in the BRCA cohort and normal breast tissues in GTEx (Mann–Whitney test, ^∗^*p* < 0.05).

The *tpd52l2* gene has at least ten transcript variants yet one protein isoform according to previous works [21, 22]. To assess which transcript was translated into protein, we first examined the expression of all ten transcript variants in cancer patients from 33 cohorts of TCGA datasets. Unsupervised clustering analyses classified 16 cancer types as a major clade, including BLCA, LUAD, READ, COAD, BRCA, and SKCM (Figure 4(a)). These cancer types displayed lower expression of transcript variants 8, 3, 1, 2, and 4 (V8, V3, V1, V2, and V4) and higher expression of transcript variants 5, 6, 10, and 9 (V5, V6, V10, and V9) than those of other cohorts. BRCA and SKCM were clustered into one subclade, indicating that breast carcinoma had the closest relationship with skin cutaneous melanoma in this analysis. There were several minor clades, including one minor clade with higher expression of V8 in KICH, HNSC, LUSC, and KIRC and another minor clade with lower expression of V10 in LIHC, DLBC, UCEC, and ACC, when compared to other cancer types (Figure 4(a)). Gene structures of the transcripts, excluding the 3′-UTR and 5′-UTR, are shown in Figure 4(b), and the predicted intron/exon phases are marked. The transcripts in the same subclades exhibited highly conserved gene structural patterns.

We identified that V5 and V6 were the top two enriched transcripts in patients using the median value of one as the expression cutoff value (Figure 4(c)). Due to the higher abundance of V5 and V6 in BRCA patients, we hypothesized that one of the two transcripts could be translated into the functional protein TPD52L2 in BLBCs. We compared the expression of V5 and V6 in breast cancer tissues and paired adjacent normal tissues according to the PAM50 classification. V5 expression showed no difference between breast cancer tissues and paired adjacent normal tissues in any subtype. In contrast, V6 expression was markedly elevated in breast cancer tissues compared with paired adjacent normal tissues in all PAM50 subtypes, except the Her2-enriched subtype (Figures 4(d) and 4(e)). We also examined the expression of each transcript between breast cancer tissues and normal breast tissues of the GTEx dataset. V6 expression was markedly elevated in tumor tissues compared with normal tissues, whereas V5 and other transcripts showed no significant difference (Figure 4(f)). These clinical results suggested that V6 is most likely to be the functional transcript of the TPD52L2 protein.

### 3.4. TPD52L2 Knockdown Suppressed the Proliferation and Migration of BLBC Cells

**Figure 5:**
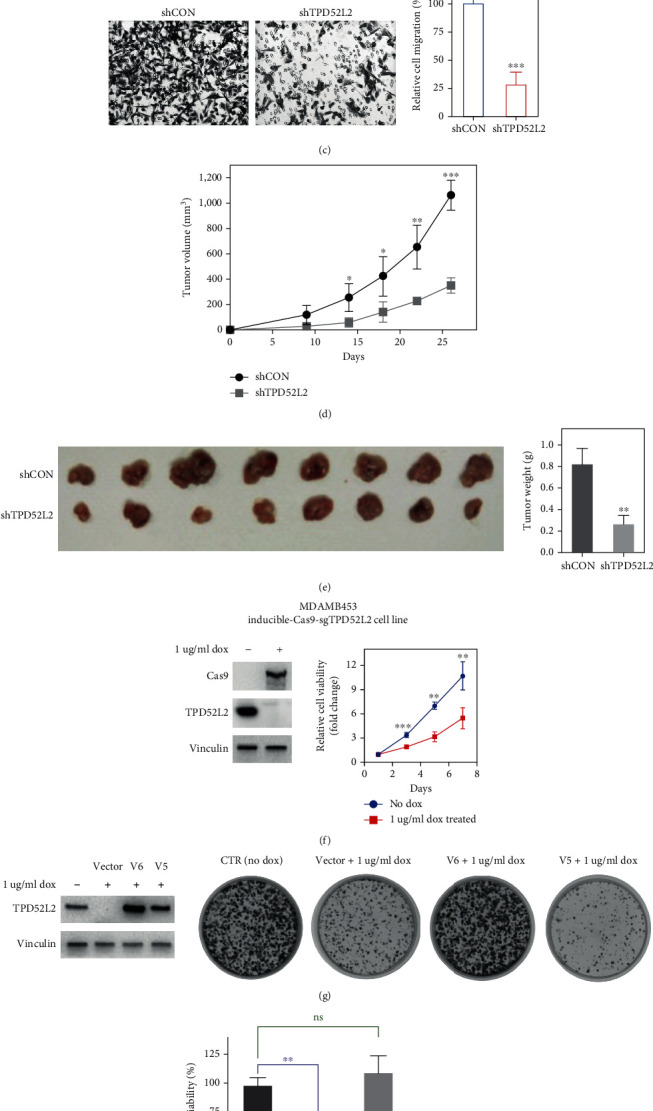
The effect of TPD52L2 on the tumorigenesis of BLBC cells *in vitro* and *in vivo*. (a–e) The MDA-MB-231 cell line was transfected with either shCON or 3 different shRNAs targeting TPD52L2. After puromycin selection and positive colony enrichment, MDA-MB-231 cell lines with stable knockdown were established. (a) Cell viability assays. The samples were assayed in triplicate. Each point represents the mean value from three independent samples. (b) Colony formation assays. (c) Cell migration assays. Representative photographs and bar graphs were from three independent experiments (mean ± SD, *n* = 3, *t*-test, ^∗^*p* < 0.05). (d) Growth curves of xenograft tumors obtained upon subcutaneous implantation of shCON or shTPD52L2 cells. Tumor volumes were monitored every 3 days by measuring tumor diameters (mean ± SD, *n* = 10, ^∗^*p* < 0.05). (e) Images and weights of xenograft tumors. The 5tumors were removed, photographed, and weighed (mean ± SD, *n* = 10, *t*-test, ^∗^*p* < 0.05). (f–i) MDA-MB-453 cells were infected with lentiviruses expressing CAS9 under the control of a doxycycline-inducible promoter and lentiviruses expressing a sgRNA targeting TPD52L2 (sgTPD52L2). Stable inducible Cas9-sgTPD52L2 cell lines were established after two rounds of selection using blasticidin and puromycin. (f) Cell viability assays of inducible Cas9-sgTPD52L2 cell lines. The samples were assayed in triplicate. Each point represents the mean value from three independent samples. (g) The stable cell lines were treated with doxycycline for 48 h and then washed and transfected with plasmids containing the cDNAs of V5 or V6 tagged with HA. Forty-eight hours after transfection, the cells were lysed and immunoblotted as indicated. (h) Colony formation assays. (i) Cell migration assays. Representative photographs and bar graphs are from three independent experiments. The data are presented as the mean ± SD (*n* = 3, *t*-test, ^∗^*p* < 0.05).

Based on the clinical data, we hypothesized that TPD52L2 could help to maintain the malignant phenotypes of BLBC cells. We selected highly aggressive basal-like MDA-MB-231 cells and established a stable knockdown cell line via lentiviral infection of TPD52L2 shRNA (Figure 5(a)) [23]. We detected a dramatic decrease in cell proliferation via the CCK-8 assay and further observed that the ability to form colonies was remarkably suppressed in TPD52L2 stable knockdown cells (Figures 5(a) and 5(b)). The colony number of knockdown cells was less than 50% of that of the control cells (Figure 5(b)). Moreover, cell migration decreased significantly after TPD52L2 knockdown (Figure 5(c)). The migration rate of control cells was approximately 4-fold higher than that of knockdown cells. No change in cell apoptosis was detected using annexin V staining and flow cytometry (data not shown).

To determine the effect of TPD52L2 on tumorigenesis *in vivo*, we subcutaneously injected control and stable knockdown cells into nude mice and monitored tumor growth. As shown in Figure 5(d), the potential to form tumors was markedly decreased in the TPD52L2 stable knockdown line when compared to the control line at the same time. Tumor volumes of the control line reached over 1100 mm^3^ in 28 days, whereas tumor volumes of the knockdown line were less than 400 mm^3^ on the 27th day. The tumor weights of the knockdown line decreased more than 3-fold compared to those of the control line (Figure 5(e)).

Taken together, our findings revealed the impact of TPD52L2 on breast cancer patient survival and responses to radiation and targeted therapies, its association with pathological stages and lymph node metastasis, its enriched expression in BLBCs, and its important roles in maintaining malignant phenotypes and promoting tumor growth, establishing TPD52L2 as a novel biomarker in BLBC.

### 3.5. V6 Was Identified as the Functional TPD52L2 Protein in BLBC

We compared the amino acid sequences of ten protein isoforms of TPD52L2. V6 had the same molecular weight as the endogenous TPD52L2 protein, further supporting our hypothesis. To corroborate and expand the results, we investigated whether V5 or V6 was required to rescue the functional loss of TPD52L2 and to maintain the cancer-related phenotypes. We produced TPD52L2 inducible knockout cells using sgRNA with doxycycline-induced CAS9 according to CRISPR-Cas9 knockout techniques (Figure 5(f)). Our attempts to produce stable TPD52L2 knockout cells were not successful, suggesting that the BLBC cell lines we tested failed to survive without TPD52L2 function. All experiments were performed with the BLBC cell line MDA-MB-453 [23].

Consistent with the results of MDA-MB-231 stable knockdown cells, inducible depletion of TPD52L2 caused a dramatic decrease in MDA-MB-453 cell proliferation and migration, the levels of which were approximately 25% and 15% of those of the uninduced control line, respectively (Figures 5(g)–5(i)). V6 markedly increased cell proliferation in the colony formation assay in TPD52L2 inducible knockout cells, which had nearly the same number and size of colonies as the control line, and there was an increasing but nonsignificant trend (Figure 5(g)). V5 exerted an inhibitory effect on cell proliferation, with an approximately 2-fold decrease compared to that in the control cells (Figures 5(g) and 5(h)). Similarly, we observed a significant increase in cell migration upon expression of V6 in TPD52L2 inducible knockout cells, and the migration rate was restored to the same level as that in uninduced control cells (Figure 5(i)). V5 had no detectable effect on migration (Figure 5(i)). Therefore, V6 was proven to be the functional isoform of the TPD52L2 protein that positively regulates the oncogenesis phenotypes of BLBC.

### 3.6. Expression of V6 Is Closely Associated with the Clinicopathological Parameters of Breast Cancer

**Figure 6:**
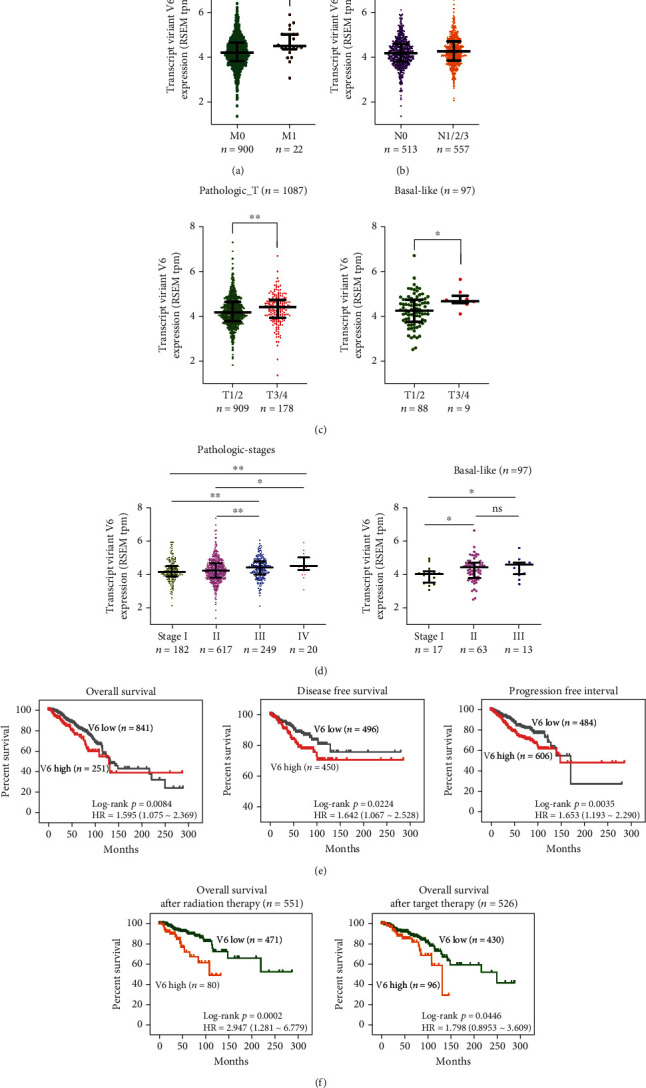
Expression of V6 of *tpd52l2* in patients with different pathological characteristics and its impact on survival and therapy response. (a) Comparative analysis of transcript expression of V6 in breast cancer patients with pathological_M0 status and pathological_M1 status. (b) Comparative analysis of transcript expression of V6 in breast cancer patients with pathological_N0 status and higher status (N1, N2, and N3). (c) Comparative analysis of transcript expression of V6 in breast cancer patients or BLBC patients with lower pathological T stage (T1 and T2) and higher pathological T stage (T3 and T4). (d) Comparative analysis of transcript expression of V6 in breast cancer patients or BLBC patients with different pathological stages (stages I, II, III, and IV). (e) Kaplan–Meier curves indicating the OS, DFS, and PFI of breast cancer patients in the BRCA cohort. (f) Kaplan–Meier curves indicating the OS of breast cancer patients who received radiation therapy or targeted therapy in the BRCA cohort (Kaplan–Meier analysis with log-rank test for survival analysis, Mann–Whitney test for expression analysis, ^∗^*p* < 0.05).

Finally, we analyzed the correlation between the expression of V6 and the clinicopathological parameters of patients in the BRCA cohort. As shown in Table 1, elevated V6 expression was strongly associated with higher pathological metastasis (pM) stage (*n* = 928, Fisher's exact test, *p* = 0.008415), higher pT stage (*n* = 1087, the *χ*^2^ test, *p* < 0.001), and worse pathological stage (*n* = 1068, Fisher's exact test, *p* < 0.001). V6 expression was significantly higher in patients with pathological_M1 stage disease than in patients with pathological_M0 stage disease (Mann–Whitney test, *p* = 0.0039) (Figure 6(a)). The median expression value of 4.506 in patients with pathological_M1 stage was significantly higher than the median expression value of 4.218 in patients with pathological_M0 stage. V6 expression was slightly lower in patients with pathological_N0 status than in those with pathological_N1/2/3 status (Mann–Whitney test, *p* < 0.05) (Figure 6(b)). Additionally, patients with high V6 expression were more likely to develop advanced pathological T stage disease (Figure 6(c)). Breast cancer patients with pathological stage III/IV disease had higher V6 transcript expression than those with stage I/II (Figure 6(d)). No significant correlation was found between V6 expression and other clinical parameters, such as age, pathological node status (pN), ER expression, PR expression, and HER2 expression (Table 1).

More importantly, in BLBC, patients with high V6 expression were more likely to exhibit advanced pathological stage and worse pT stage disease than those with luminal A, luminal B, and Her 2-enriched subtypes (Figures 6(c) and 6(d)). V6 was increased in patients with pT3/4 stage disease compared with those with pT1/2 stage disease (Mann–Whitney test, *p* = 0.0145) (Figure 6(c), Table 2) and increased in parallel with pathological stage (stage I *vs.* stage II and stage III) (Figure 6(d), Table 2).

Furthermore, V6 was found to be a prognostic indicator for BRCA patients. Its elevated expression was closely associated with poor survival (Figure 6(e)). The overall survival of BRCA patients who received radiation therapy or targeted molecular therapy was closely correlated with V6 expression. Patients in the high V6 group had worse outcomes after radiation therapy or target molecular therapy than patients in the low V6 group (Kaplan–Meier survival analysis with log-rank test, radiation therapy: *p* = 0.0002; target therapy: *p* = 0.046) (Figure 6(f)).

## 4. Discussion

The well-known characteristics of BLBC include poor differentiation, squamous metaplasia (skin-like differentiation with or without sebaceous elements), lack of hormone receptor expression, high proliferation capacity, and expression of basal CKs [24]. More effective treatments and methods for identifying earlier-stage disease are urgently needed for this subtype. In the present study, we provided new clinical evidence that TPD52L2 is significantly upregulated in BLBCs versus non-BLBCs and that its high expression is closely associated with a poor prognosis in patients with BLBC. The finding that high TPD52L2 expression is closely related to advanced pathological stage may imply a relationship between TPD52L2 and tumorigenesis (Figure 1(f)). Generally, tumors of higher pathological stages grow and metastasize more rapidly than tumors of lower pathological stages. Therefore, the correlation between TPD52L2 expression and tumor pathological stage supports our study on the growth- and metastasis-promoting effects of TPD52L2 in BLBC cell lines. Furthermore, higher TPD52L2 expression was significantly predictive of worse clinical outcome in lymph node-positive patients with BLBC, indicating a potential relationship between TPD52L2 and malignancies with lymph node metastasis. A previous study reported that TPD52 was a radiation response biomarker in several cancer cell lines [3]. In our study, TPD52L2 gene expression was also proven to be closely associated with the efficacy and outcome of radiation therapy, as breast cancer patients with low TPD52L2 levels had longer survival times after radiation therapy, as well as target therapy, than those with high TPD52L2 levels (Figures 3(e) and 3(f)). Since our study mainly investigated the RNA level of TPD52L2 in patients with BLBC, a detailed clinical study of patients' TPD52L2 protein level by IHC staining is required to verify the relationship between TPD52L2 and tumorigenesis, and further studies are needed if that is the case. Overall, our clinical analyses demonstrated for the first time that TPD52L2 is closely associated with the development and progression of BLBC and suggests that TPD52L2 is a biomarker for BLBC patients.

Zhuang et al. showed that TPD52L2 overexpression inhibited colony formation and cell proliferation in luminal-type MCF7 cells [23]. Here, we presented new clinical evidence that TPD52L2 had no correlation with the prognosis of patients with luminal A and luminal B breast cancers and failed to predict the clinical outcomes of lymph node-positive patients with luminal subtype breast cancers (Figures 2(a) and 2(b)). Therefore, based on the positive correlation between TPD52L2 and the clinical parameters of BLBC, in our study, we examined the effects of TPD52L2 on the tumorigenesis of MDA-MB-231 and MDA-MB-453 cells and tumors derived from these cells. We found that decreased TPD52L2 expression by knockdown or knockout significantly inhibited cell growth *in vitro* and *in vivo*, and this inhibitory effect could be mainly attributed to TPD52L2-mediated positive regulation of cell proliferation. A currently accepted model for TPD52L2 cellular function involves its recognition of binding partners, including TPD52, TPD53, and several Rab GTPases, as well as its involvement in multiple membrane trafficking pathways: anterograde traffic, recycling, and Golgi integrity [11]. It is possible that TPD52L2 promotes BLBC progression partially due to its important roles in intracellular transport vesicles. However, the detailed molecular mechanisms in BLBC still need to be investigated in depth. Most recently, TPD52L2 was reported to be involved in cell migration, as it is a core protein of intracellular nanovesicles, in which it was proven to mediate *α*5*β*1 integrin trafficking during the migration process [25]. Consistently, in the present study, we not only provided clinical evidence that patients with BLBC with high TPD52L2 levels had a significantly poorer prognosis than those with low levels but also provided novel experimental results that depletion of TPD52L2 inhibited cell migration, while overexpression of V6 reversed this inhibition in BLBC cells. This finding further supports that TPD52L2 is involved in the malignant phenotypes of human cancers.

Over the years, increasing evidence has shown that TPD52 family members have many transcript variants due to alternative splicing [22, 23, 26]. Different transcripts of a gene generated by alternative splicing are often translated into proteins with altered domains of composition, thus affecting their biological functions. The well-known transcription factor SREBP1, which mainly controls fatty acid synthesis, has two protein isoforms, SREBP1-a and SREBP1-c. They were generated from the same gene, *srebf1*, by posttranscriptional alternative splicing [27]. TPD52L2 has at least 10 transcript variants in human tissues [24]. To date, there is no compelling evidence about the transcript variant of TPD52L2 that plays the most important role in BLBC. In the present study, we utilized a two-step process to investigate the functional protein isoform of TPD52L2 in breast cancer. First, we analyzed the expression profile of ten transcript variants of *tpd52l2* in different cancer types from 33 cohorts of TCGA dataset via hierarchical clustering and verified two predominantly expressed transcript variants, V5 and V6, in breast cancer and several other cancer types (Figure 4(a)). Interestingly, in the present study, BRCA and SKCM were clustered into one subclade, proving that the expression profile of *tpd52l2* is associated with the squamous metaplasia characteristics of BLBCs. Moreover, a previous study proved that BRCA and SKCM have the most similar RNA expression patterns of all metastasis-associated genes among 11 cancer types in TCGA dataset [28]. We can infer that, to some extent, BRCA and SKCM share similarities in metastasis-related phenotypes and mechanisms. Next, we identified cancer-specific transcripts according to relative transcript levels between normal and cancer tissues and corroborated that V6 was the breast cancer-specific transcript. The specificity of V6 was further confirmed by comparing the molecular weights and amino acid sequences of all protein isoforms encoded by the ten transcript variants of the *tpd52l2* gene. Moreover, we overexpressed V5 and V6 in TPD52L2-depleted BLBC cells and excluded the possibility of V5, which failed to promote cell proliferation and migration (Figures 5(g) and 5(i)). Accordingly, using the clinical data from the BRCA cohort, we observed that the V6 transcript was remarkably upregulated in cancer tissues compared with paired adjacent normal tissues, and its high level was significantly associated with higher pathological T stage and pathological stage in BLBCs. These data further support the potential impact of TPD52L2 on cancer-related phenotypes (Figures 6(a)–6(d)). We also found that V6 expression could predict the prognosis of patients with SKCM and patients with MESO receiving radiation therapy or targeted therapy (data not shown). This result further supported the close relationship of BRCA and SKCM or MESO in the hierarchical clustering generated by the transcript expression profile of *tpd52l2* and provided solid evidence that V6 plays oncogenic roles in SKCM and MESO. Most recently, a detailed bioinformatic analysis revealed the oncogenic role of TPD52L2 in lung adenocarcinoma and demonstrated that its high expression is associated with immune infiltration and tumor immunosuppressive status, further supporting our results [29].

In summary, our current findings demonstrated that V6 of TPD52L2 is a novel biomarker for the prediction of clinical outcomes in BLBCs. V6 promotes cell proliferation and migration and has a close association with malignant phenotypes in BLBCs. Due to the association of V6 expression and patient prognosis after radiation therapy or targeted therapy in SKCM and MESO datasets, we recommend that further studies address whether TPD52L2 plays a similar malignancy-related role in other cancer types. More importantly, since the coiled-coil domain has been identified as an effective drug target for several severe disease treatments [30], our data indicated that TPD52L2 can be used as an effective biomarker and a therapeutic target for BLBC. Further investigations are required to achieve the clinical application, relieve patients' suffering, and overcome those incurable diseases.

## Figures and Tables

**Table 1:** Association between the transcript expression of V6 and clinicopathological parameters in breast cancer patients of the BRCA cohort.

Clinicopathological parameters	*n*	TPD52L2 expression		*p* value
		High (>median)	Low (<median)	
Age				
≥60	370	169	201	0.8141
≤60	443	206	237	
Her2				
Positive	113	59	54	0.1575
Negative	647	288	359	
PR				
Positive	518	226	292	0.05107
Negative	253	130	123	
ER				
Positive	597	269	328	0.3822
Negative	177	87	90	
Pathologic_M				
M0	906	435	471	0.008415^∗^
M1	22	17	5	
Pathologic_N				
N0	513	244	269	0.05549
N1	361	171	190	
N2	119	71	48	
N3	77	43	34	
Pathologic_T				
T1	280	114	166	0.000301^∗^
T2	629	319	310	
T3	138	85	53	
T4	40	24	16	
Pathologic_stage				
Stage I	182	76	106	0.0006917^∗^
Stage II	617	297	320	
Stage III	249	144	105	
Stage IV	20	15	5	

Age: at initial pathological diagnosis; pTNM: pathological tumor node metastasis; pT: pathological tumor; pN: pathological node. The median expression value was 4.251. ^∗^The significance of correlations between V6 expression and clinicopathological parameters was calculated by the *χ*^2^ test, and Fisher's exact test was used when the patient number had an expected count < 5.

**Table 2:** Association between the transcript expression of V6 and clinicopathological parameters in patients with BLBC.

Clinicopathological parameters	*n*	TPD52L2 expression		*p* value
		High (>median)	Low (<median)	
Pathologic_T				
T1	22	6	16	0.0099^∗^
T2	65	35	30	
T3	8	7	1	
T4	1	1	0	
Pathologic_M				
M0	94	48	46	1
M1	3	2	1	
Pathologic_stage				
Stage I	17	3	14	0.0101^∗^
Stage II	63	35	28	
Stage III	13	9	4	
Stage IV	2	1	1	
Pathologic_N				
N0	61	29	32	0.6541
N1	24	13	11	
N2	8	5	3	
N3	4	3	1	

Age: at initial pathological diagnosis; pTNM: pathological tumor-node-metastasis; pT: pathological tumor; pN: pathological node. The median expression value was 4.251. ^∗^The significance of correlations between V6 expression and clinicopathological parameters was calculated by the *χ*^2^ test, and Fisher's exact test was used when the patient number had an expected count < 5.

## Data Availability

TCGA data is openly available in the public repository: https://xenabrowser.net. Kaplan–Meier data of the GSE96058 dataset is openly available in the published resource: http://kmplot.com/. Other data will be available on reasonable request from the authors.

## Abbreviations

BC: Breast cancer
BLBC: Basal-like breast cancer
TCGA: The Cancer Genome Atlas
ACC: Adrenocortical carcinoma
BLCA: Bladder urothelial carcinoma
BRCA: Breast invasive carcinoma
CESC: Cervical squamous cell carcinoma
CHOL: Cholangiocarcinoma
COAD: Colon adenocarcinoma
READ: Rectum adenocarcinoma
DLBC: Diffuse large B-cell lymphoma
ESCA: Esophageal carcinoma
GBM: Glioblastoma multiforme
HNSC: Head and neck squamous cell carcinoma
KICH: Kidney chromophobe
KIRC: Kidney renal clear cell carcinoma
KIRP: Kidney renal papillary cell carcinoma
AML: Acute myeloid leukemia
LGG: Brain lower grade glioma
LIHC: Liver hepatocellular carcinoma
LUAD: Lung adenocarcinoma
LUSC: Lung squamous cell carcinoma
MESO: Mesothelioma
OVSC: Ovarian serous cystadenocarcinoma
PAAD: Pancreatic adenocarcinoma
PCPG: Pheochromocytoma and paraganglioma
PRAD: Prostate adenocarcinoma
SARC: Sarcoma
SKCM: Skin cutaneous melanoma
STAD: Stomach adenocarcinoma
TGCT: Testicular germ cell tumor
THCA: Thyroid carcinoma
THYM: Thymoma
UCEC: Uterine corpus endometrial carcinoma
UCS: Uterine carcinosarcoma
UVM: Uveal melanoma
SREBP1: Sterol regulatory element binding protein 1
INVs: Intracellular nanovesicles.

## References

[1] Wilkinson L., Gathani T. (2022). Understanding breast cancer as a global health concern. *The British Journal of Radiology*.

[2] Banerjee S., Reis-Filho J. S., Ashley S. (2006). Basal-like breast carcinomas: clinical outcome and response to chemotherapy. *Journal of Clinical Pathology*.

[3] Gusterson B., Eaves C. J. (2018). Basal-like breast cancers: from pathology to biology and back again. *Stem Cell Reports*.

[4] Milioli H. H., Tishchenko I., Riveros C., Berretta R., Moscato P. (2017). Basal-like breast cancer: molecular profiles, clinical features and survival outcomes. *BMC Medical Genomics*.

[5] Pollack J. R., Sørlie T., Perou C. M. (2002). Microarray analysis reveals a major direct role of DNA copy number alteration in the transcriptional program of human breast tumors. *Proceedings of the National Academy of Sciences of the United States of America*.

[6] Rubin M. A., Varambally S., Beroukhim R. (2004). Overexpression, amplification, and androgen regulation of TPD52 in prostate cancer. *Cancer Research*.

[7] Kamili A., Roslan N., Frost S. (2015). TPD52 expression increases neutral lipid storage within cultured cells. *Journal of Cell Science*.

[8] Abe Y., Mukudai Y., Kurihara M. (2021). Tumor protein D52 is upregulated in oral squamous carcinoma cells under hypoxia in a hypoxia-inducible-factor-independent manner and is involved in cell death resistance. *Cell & Bioscience*.

[9] Delaidelli A., Dunham C., Santi M. (2022). Clinically tractable outcome prediction of non-WNT/non-SHH medulloblastoma based on TPD52 IHC in a multicohort study. *Clinical Cancer Research*.

[10] Hein M. Y., Hubner N. C., Poser I. (2015). A human interactome in three quantitative dimensions organized by stoichiometries and abundances. *Cell*.

[11] Larocque G., La-Borde P. J., Clarke N. I., Carter N. J., Royle S. J. (2020). Tumor protein D54 defines a new class of intracellular transport vesicles. *The Journal of Cell Biology*.

[12] Goldman M. J., Craft B., Hastie M. (2020). Visualizing and interpreting cancer genomics data via the Xena platform. *Nature Biotechnology*.

[13] Vivian J., Rao A. A., Nothaft F. A. (2017). Toil enables reproducible, open source, big biomedical data analyses. *Nature Biotechnology*.

[14] Lánczky A., Győrffy B. (2021). Web-based survival analysis tool tailored for medical research (KMplot): development and implementation. *Journal of Medical Internet Research*.

[15] Zhang X., Zhao Y., Wang C. (2018). Rhomboid domain-containing protein 1 promotes breast cancer progression by regulating the p-Akt and CDK2 levels. *Cell Communication and Signaling: CCS*.

[16] Metsalu T., Vilo J. (2015). ClustVis: a web tool for visualizing clustering of multivariate data using principal component analysis and heatmap. *Nucleic Acids Research*.

[17] Hu B., Jin J., Guo A. Y., Zhang H., Luo J., Gao G. (2015). GSDS 2.0: an upgraded gene feature visualization server. *Bioinformatics*.

[18] Sievers F., Wilm A., Dineen D. (2011). Fast, scalable generation of high-quality protein multiple sequence alignments using Clustal Omega. *Molecular Systems Biology*.

[19] Saleh M. M., Scheffler M., Merkelbach-Bruse S. (2022). Comprehensive analysis of TP53 and KEAP1 mutations and their impact on survival in localized- and advanced-stage NSCLC. *Journal of Thoracic Oncology*.

[20] Parker J. S., Mullins M., Cheang M. C. (2009). Supervised risk predictor of breast cancer based on intrinsic subtypes. *Journal of Clinical Oncology*.

[21] Qiang Z., Jun-Jie L., Hai W. (2018). TPD52L2 impacts proliferation, invasiveness and apoptosis of glioblastoma cells via modulation of wnt/*β*-catenin/snail signaling. *Carcinogenesis*.

[22] Zhuang Y., Ly R. C., Frazier C. V. (2019). The novel function of tumor protein D54 in regulating pyruvate dehydrogenase and metformin cytotoxicity in breast cancer. *Cancer & Metabolism*.

[23] Neve R. M., Chin K., Fridlyand J. (2006). A collection of breast cancer cell lines for the study of functionally distinct cancer subtypes. *Cancer Cell*.

[24] Cunningham F., Allen J. E., Allen J. (2022). Ensembl 2022. *Nucleic Acids Research*.

[25] Niu N., Qin Y., Fridley B. L. (2010). Radiation pharmacogenomics: a genome-wide association approach to identify radiation response biomarkers using human lymphoblastoid cell lines. *Genome Research*.

[26] Fan Y., Hou T., Gao Y. (2021). Acetylation-dependent regulation of TPD52 isoform 1 modulates chaperone-mediated autophagy in prostate cancer. *Autophagy*.

[27] Horton J. D., Goldstein J. L., Brown M. S. (2002). SREBPs: activators of the complete program of cholesterol and fatty acid synthesis in the liver. *The Journal of Clinical Investigation*.

[28] Chen F., Zhang Y., Varambally S., Creighton C. J. (2019). Molecular correlates of metastasis by systematic pan-cancer analysis across The Cancer Genome Atlas. *Molecular Cancer Research*.

[29] Zhong A., Chen T., Zhou T., Zhang Z., Shi M. (2021). TPD52L2 is a prognostic biomarker and correlated with immune infiltration in lung adenocarcinoma. *Frontiers in Pharmacology*.

[30] Strauss H. M., Keller S. (2008). Pharmacological interference with protein-protein interactions mediated by coiled-coil motifs. *Handbook of Experimental Pharmacology*.

